# Taking it to the bank: the ethical management of individual findings arising in secondary research

**DOI:** 10.1136/medethics-2020-106941

**Published:** 2021-01-13

**Authors:** Mackenzie Graham, Nina Hallowell, Berge Solberg, Ari Haukkala, Joanne Holliday, Angeliki Kerasidou, Thomas Littlejohns, Elizabeth Ormondroyd, John-Arne Skolbekken, Marleena Vornanen, The Northern European Returning Results Network, Naomi Allen

**Affiliations:** 1 Wellcome Centre for Ethics and Humanities, University of Oxford, Oxford, UK; 2 Ethox Centre, Nuffield Department of Population Health, University of Oxford, Oxford, UK; 3 Department of Public Health and General Practice, The Norwegian University of Science and Technology, Trondheim, Norway; 4 Faculty of Social Sciences; Helsinki Collegium for Advanced Studies, University of Helsinki, Helsinki, Finland; 5 Nuffield Department of Population Health, University of Oxford, Oxford, UK; 6 NIHR Oxford Biomedical Research Centre, University of Oxford, Oxford, UK; 7 Center for Population, Health and Society, University of Helsinki, Helsinki, Finland; 8 Open University, Milton Keynes, UK

**Keywords:** ethics, research ethics, genethics

## Abstract

A rapidly growing proportion of health research uses ‘secondary data’: data used for purposes other than those for which it was originally collected. Do researchers using secondary data have an obligation to disclose individual research findings to participants? While the importance of this question has been duly recognised in the context of primary research (ie, where data are collected from participants directly), it remains largely unexamined in the context of research using secondary data. In this paper, we critically examine the arguments for a moral obligation to disclose individual research findings in the context of primary research, to determine if they can be applied to secondary research. We conclude that they cannot. We then propose that the nature of the relationship between researchers and participants is what gives rise to particular moral obligations, including the obligation to disclose individual results. We argue that the relationship between researchers and participants in secondary research does not generate an obligation to disclose. However, we also argue that the biobanks or data archives which collect and provide access to secondary data may have such an obligation, depending on the nature of the relationship they establish with participants.

## Introduction

The challenges presented by the feedback of individual research findings are well recognised in the bioethics literature. If a researcher discovers something of possible clinical significance during their research, but which is beyond the aims of the study, (ie, an ‘incidental finding’) to whom should this be disclosed, if anyone? Do researchers have an obligation to actively seek out potentially clinically significant information that is unrelated to the primary purpose of testing (ie, ‘secondary findings’), and should every such finding receive expert analysis to assess its clinical significance? Should participants be provided with any information regarding their individual-level research results, and what should research ethics committees require regarding feedback?

To date, much of the discussion of feeding back research findings has focused on a specific research arrangement, what we call ‘primary research’. In primary research, a researcher collects data directly from participants (eg, biological samples, information, images, etc) with the expressed purpose of answering a specific research question or set of questions. In most cases, the participant provides some form of informed consent, confirming that, inter alia, they understand the purposes of the research.

However, primary research is not the only context in which questions about the feedback of research findings can arise. A significant proportion of current research uses data drawn from secondary sources such as biobanks or data archives and is conducted by researchers that have no contact with the individual patients or participants who provided the data. What obligations, if any, do these ‘secondary researchers’ have to feed back research findings and what is the basis of this obligation?

## Collecting and storing research data: biobanks and data archives

Biobanks are growing in popularity as a source of research data. Participants in a biobank voluntarily undergo a battery of tests as part of an observational study and allow the resultant data to be stored by the biobank for use in future research. Data curators process raw participant data on behalf of the biobank, including identifying incorrect or incomplete data, and harmonising data from different sources, to ensure they are useable for research. In some cases, this research will be conducted by researchers who are a part of the biobank, and in other cases, the biobank will grant access to this data to outside researchers. At the time the data are collected, participants are not specifically told what their data will be used for. In most cases, researchers who will eventually use these data do not have any contact with the participants who contributed their data and may only receive data stripped of any possible identifiers (ie, ‘deidentified’). The Northern European Returning Results Network is a group of bioethicists, social scientists, epidemiologists, and clinical researchers who are associated with a number of these types of studies (box 1).

Box 1 **The Northern European returning results network**

UK Biobank is a long-term observational study which makes available a wide range of health-related data, including genetic and biomarker data measured from blood and urine samples, as well as whole-body imaging data, to external researchers.^47 48^ Similarly, the National FINRISK Study, a large longitudinal population survey (1972–2012) in Finland, has collected data on risk factors in chronic non-communicable diseases, and recently expanded to collect genotypic and other biomarker data collection on a subset of the population.^49^ Data from the FINRISK study is used for various research activities and national health monitoring. Finally, Norway’s Nord Trøndelag Health Study was carried out in four phases between 1984 and 2019, and used questionnaires, interviews, clinical studies and analysis of blood, saliva and urine samples to generate a biobank of more than 126 000 unique participants.^50^

Researchers might also access research data through a data archive. Unlike a biobank, a data archive does not collect data from participants directly; it acts as a repository for research data already collected through primary research or clinical practice. For example, the UK’s National Consortium of Intelligent Medical Imaging (NCIMI) and PathLAKE are repositories for annotated data of medical images and digitised pathology slides, respectively, collected from National Health Service patients. Similarly, the Mount Sinai Health System in New York collects clinical images from over 1 million patients, and integrates them with electronic health records, in its Imaging Research Warehouse. Researchers accessing data from a data archive do not typically have any contact with the original participants from whom the data were collected, and only receive deidentified datasets. Moreover, because regulations such as the General Data Protection Regulations (GDPR) in the European Union (EU) permit the processing of patient health records for research purposes without consent, patients may be unaware of the specific nature of the research for which their data are being used, or even that their data are being used at all.

Regulations governing the processing of personal data, including health data, may vary significantly by jurisdiction. In the UK and EU, identifiable data produced in the course of clinical care may be used for research purposes without the informed consent of the patient, provided it meets one of several legal bases for processing, including being necessary for the performance of a public task, a legal obligation, or legitimate interests. Even when informed consent is not the legal basis for data use, patients have the right to be informed about the collection and use of their personal data.^1^ Additionally, in the UK, all health and social care organisations which make use of patient health and care information must have a Caldicott Guardian, a senior person responsible for ensuring that personal data is used legally and ethically.^2^ The national data opt-out allows individuals to opt-out of the use of their personal data.

Conversely, in the USA, the processing of personal health information is governed by the Health Insurance Portability and Accountability Act, but only applies to data created or held by ‘covered entities’, including health plans, provider organisations and clearinghouses, and their subcontractors.^3^ Thus, health information collected by commercial companies (eg, from fitness trackers or other ‘health apps’)—which may be used for secondary research—are not subject to this regulation.

In this paper, we refer to data which have been collected by or on behalf of someone other than the user as ‘secondary data.’ Participants who contribute their data to a biobank, or whose data are accumulated in a data archive, contribute secondary data and are referred to as ‘data contributors’. A participant in primary research becomes a ‘data contributor’ once their data are made accessible to researchers outside the study for which they were originally collected. Likewise, we refer to any research using secondary data from data contributors as ‘secondary research’, and the researchers making use of such data as ‘secondary researchers.’ For example, a researcher using saliva samples collected from the Trøndelag Health Study, or clinical data from the FINRISK study, is a secondary researcher. A researcher employed by UK Biobank who is conducting research on genomic markers of Alzheimer’s disease, using blood samples collected by the biobank, is also a secondary researcher, because the data they are using were not collected specifically for this purpose. (Thus, there is no primary researcher in this case). Conversely, a researcher who collects genomic data from participants to study the genetics of learning disorders is a primary researcher, but if these data are subsequently deposited in a data archive, a researcher who accesses the deposited data is a secondary researcher. For example, researchers in the SCARFE (Secondary Cardiac Findings Evaluation) study are exploring the pathogenicity of genomic variants associated with cardiac disease in individuals whose genomic data are contained in the NIHR BioResource for Rare Disease.^4^ Finally, a radiographer at a biobank who acquires an MRI scan from a data contributor and analyses the scan for image quality is not a researcher at all, because she is curating data and not conducting research. (Were the researcher acquiring the scans in the context of a specific research study, this would be primary research). We will consider these cases—and the responsibilities of biobanks as data curators—separately.

There are several important differences between primary and secondary research, which may influence the obligations of secondary researchers regarding the provision of individual findings. Centrally, the different relationship between the secondary researcher and data contributor (compared with primary researcher and participant) suggests that the standard justifications for disclosing potentially clinically significant findings in primary research (ie, the duty of easy rescue or beneficence, respect for persons, ancillary care obligations) may not apply in the same way to secondary research. In what follows, we examine the potential moral obligations of secondary researchers to data contributors, specifically as they relate to feeding back individual research findings. We also consider the role of intermediaries such as biobanks, from whom secondary researchers acquire data.

## What are individual research findings?

Biomedical research can generate several different types of findings. Aggregate research results offer a summary of the study’s overall findings, such as the percentage of a study population that carries a certain genetic marker associated with heart disease. In the context of primary research, offering aggregate results to participants once they have undergone peer review is generally accepted as good research practice.^5 6^ Making such results available can demonstrate gratitude for the participant’s contribution and build trust in the research enterprise. We suggest that similar considerations apply to the context of secondary research. For example, a biobank might make aggregate results of research conducted using biobank data available on its website. Because the feedback of aggregate results is not our main concern in this paper, we will not discuss these kinds of results further.

Individual research results may be termed ‘primary findings’ when they relate to the outcome measure of the research, such as whether participant A has specific changes in their heart in a cardiac imaging study. Conversely, ‘secondary findings’ are individual findings that are not the primary target of a test or procedure but may be considered appropriate for return because they may have clinical relevance for the participant. In some cases, secondary findings are actively sought: the UK 100 000 Genomes Project offers participants, regardless of the health condition for which they enrolled, feedback of pathogenic genomic variants associated with health conditions that have a long asymptomatic or presymptomatic phase and for which treatments or enhanced screening have clinical utility. This includes some genes associated with predisposition to cancer, and familial hypercholesterolaemia.^7^

Secondary findings may also be unsought, and in such cases are referred to as ‘incidental findings.’ Incidental findings are sometimes further divided into ‘anticipatable’ or ‘unanticipatable’, where the former refers to findings which are known to be associated with a certain test or procedure (while not being deliberately sought), and the latter refers to findings which are not. The salient difference here is that a participant can be informed of the possibility of anticipatable incidental findings prior to participating in the study. For example, an asymptomatic brain infarct would be an anticipatable finding on a brain MRI (as these are present in 7.2% of healthy volunteers).^8^

Secondary findings and incidental findings are not uncommon. For example, research suggests that secondary genomic findings in a curated gene list are detected in approximately 3% of individual genomic sequences across a variety of settings (eg, primary care, biobanks),^9^ while incidental findings occur in 15%–89% of study contributors receiving CT colonography, and between 13% and 84% of brain scans.^10–13^

The feedback of individual research findings—primary research results, secondary findings or incidental findings—in secondary research presents many of the same ethical questions as in primary research, which have been discussed extensively: is there an obligation to return individual findings to data contributors, and if so, whose responsibility is it?^14–16^ But secondary research presents an added layer of complexity.

First, the introduction of additional parties changes the nature of the relationship between the data contributor and the researchers using their data, and complicates the assignment of responsibility for disclosure. If a secondary researcher detects a potential anomaly in a sample or sequence they receive from a biobank or a data archive, for example, it is unclear if it should be disclosed, and if so, who bears responsibility for disclosing this finding. Indeed, multiple secondary researchers may independently detect the same finding. Understanding their clinical significance may require further clinical evaluation,^4 17^ and in the case of some genetic findings, individual results might imply a risk to relatives.^18^ It may be unclear whether such clinical follow-up and, if appropriate, family screening, should be undertaken as a clinical or research activity.

Second, the degree and type of deidentification of the data will determine which parties have the capacity to recontact data contributors about individual findings, and if necessary, facilitate follow-up (eg, further consultation or investigations). If secondary researchers are provided with deidentified data, they will be unable to contact data contributors directly. Conversely, a biobank will have the capacity to ‘reidentify’ contributor data by linking it with identifying information (eg, contributor’s name and address), and contact a participant’s physician in the case of a finding of potential clinical significance.

Third, the potential harms and benefits of disclosure may be less straightforward in the case of secondary research. The potential time delay between acquisition of the data and generating individual research findings may impact the pertinence of the finding to the participant’s health (ie, an MRI scan taken 1 year before may not be an accurate representation of the data contributor’s current health status), as well as how receptive the data contributor may be to being recontacted (eg, a data contributor might react differently to being recontacted 3 years after originally contributing their data, compared with 3 weeks). Indeed, the data contributor may now be deceased, but the individual findings relevant for family members.^18 19^

## Who bears responsibility for determining if individual findings are returned?

There is broad agreement that primary researchers have an obligation to plan for the management of individual research findings, and that participants have a right to have this procedure clearly conveyed to them as part of the informed consent process.^20–23^ However, there remains significant debate in the bioethics and clinical research community about what the content of these plans should be,^7 24 25^ and feedback policies may differ considerably between research labs.^26 27^

The structural features of secondary research imply that any feedback of individual findings must be mediated by the biobank or data archive, or the primary researcher. The biobank or data archive controls access to the data, as well as the means of recontact, if possible, meaning they are responsible for setting the terms of use. Contributors also report preferring the feedback of results to occur via a person they are familiar with^28–30^—which could either be the primary researcher who enrolled the contributor or collected the data, the data contributor’s primary physician or a representative of the biobank.

## How should individual research findings arising through secondary research be managed?

Several strategies for managing individual research findings might be adopted by biobanks or data archives. They could adopt a blanket policy of non-disclosure, with no return of any individual findings regardless of clinical actionability. Such a decision might be motivated by a desire to avoid any psychological harms to contributors (eg, anxiety or confusion arising from individual research results or incidental findings whose clinical utility is unclear)^31^ and avoid confusion about the non-therapeutic nature of research participation. Scientific validity is also an important consideration; disclosing provisional individual results or incidental findings may cause data contributors to systematically change their behaviours going forward, potentially confounding the data collected in longitudinal studies, or even influencing decisions to take part. Finally, practical constraints such as a lack of resources for processing individual findings or providing necessary support for data contributors after disclosure, may be relevant.

Alternatively, individual research findings might be disclosed based on certain thresholds of clinical significance and actionability, such as when a condition is life-threatening and easily treatable.^10 32 33^ These standards might be explicitly stated (eg, by providing a list of imaging abnormalities or genes in which pathogenic variants will be disclosed) or be left to the judgement of the individual collecting/analysing the data, or another expert (eg, a radiologist). However, it may be unclear whether individual research results are clinically useful, or if the grounds for disclosure should go beyond medical benefit and include a participant’s wider interests. Disclosing individual research findings may also cause anxiety to data contributors or lead to unnecessary (and potentially costly) follow-up consultations, investigations and treatment. Thus, the net benefits of disclosure are often uncertain. On the other hand, research suggests that many research participants want to have individual research findings made available to them. (For an overview, see Shalowitz and Miller).^34^ Any feedback policy will need to account for the potential benefits and harms to data contributors, their preferences for feedback, as well as the costs to researchers (and, ultimately, the health system) of analysing and feeding back results.

## Arguments in favour of a duty to disclose individual research findings

It is a further question how these management strategies are justified. More specifically, do secondary researchers have moral obligations to data contributors which must be reflected in the feedback policy of a biobank or data archive? Do biobanks or data archives themselves have moral obligations to data contributors? It is to these questions that we now turn.

### The duty of easy rescue

Several arguments have been offered to justify disclosing at least some individual research findings in the context of primary research. However, they should not be assumed to apply automatically to secondary research. One such argument is the general duty of easy rescue.^35 36^ The basic idea is that if one can prevent something bad happening to another person by making only a slight sacrifice, one ought to prevent the harm. The duty to warn is sometimes thought of as a more specific case of the duty of easy rescue, insofar as warning someone of a potential harm usually requires little effort. Accordingly, this duty justifies the disclosure of individual research findings when the potential harm prevented is significant, and the burden on the rescuer is small. Individual research findings which are clinically significant and actionable—such as a malignant brain tumour that is easily operable, or a genetic risk that may be ameliorated by early detection (eg, cancer), or lifestyle changes (eg, long QT syndrome)—and where the contributor can be easily contacted, are examples where individual results ought to be made available.

However, procedural differences between primary and secondary research complicate applying the duty of easy rescue. The process of recontacting data contributors to ‘warn them’ about potentially significant individual research findings may be much more difficult (if it is even possible), compared with primary research. Secondary researchers may be geographically removed from data contributors, and significant time may have passed since the initial collection of the data, making recontact more difficult logistically. Relatedly, the contributor’s health status may have changed since their data were collected, making the determination of its clinical significance more difficult. The contributor might have already become aware of the finding through a clinical investigation or population screening (eg, familial hypercholesterolaemia), or alternatively, would not welcome being informed of a potential finding months or years after the data collection. Combining the challenges of reidentification and recontact (where this is even possible), with the uncertainty of whether disclosure will actually provide any benefit to the contributor, the duty of easy rescue is unlikely to justify the disclosure of individual research findings in many cases of secondary research.

### Respect for persons and reciprocity

Other approaches ground the responsibilities of researchers to disclose individual research findings in the ethical requirement of respect for persons^37^ and reciprocity.^38 39^ Research participants provide a benefit to researchers through their willingness to participate in research, and researchers ought to reciprocate this benefit through the exchange of health information which might benefit the participant. Providing information derived from research (eg, individual research findings), particularly when this information can be used to inform their future decision-making, is also argued to show respect for the data contributor as a person with their own values and goals.

A general problem with the notion of respect for persons grounding an obligation to disclose individual research findings is that many findings are themselves of unknown or uncertain clinical significance. In the absence of clear information to convey to the contributor, disclosing an incidental finding does not promote their autonomous decision making, if it leads to mistaken assumptions about their health status, for example. In cases where it is unclear if a data contributor would like to be re-contacted about individual findings, (eg, when data acquired in a care setting is subsequently used for secondary research), simply making it known that such findings exist risks contravening their ability to self-determine. This particular issue is likely to be exacerbated in secondary research, where data provenance may be unknown to the secondary researcher.

A policy of non-disclosure of individual research findings does not necessarily demonstrate a lack of respect. If a data contributor understands that a biobank will not disclose individual research findings arising from the secondary use of contributor data, and nevertheless chooses to contribute, the ends of the contributor are respected. Accordingly, we argue that respect for persons does not imply a general obligation to disclose individual research findings, for biobanks or secondary researchers.

Similarly, reciprocity does not obligate secondary researchers or biobanks to disclose individual research findings. It is worth highlighting two important features of reciprocity. The first is proportionality: what I give in return should be roughly proportionate to what I have received. The second is voluntariness: when I reciprocate what I’ve been given, I must do so freely.^40^ In this way, reciprocity differs from a contractual agreement, where a legally enforceable obligation is created. Reciprocity requires a degree of trust between the parties, insofar as it is the kind of moral attitude expected in a trust relationship: I trust that my gift will be proportionately reciprocated, and others similarly trust me to reciprocate.^41^ Contrast this again with a contract, where the conditions of the arrangement are strictly determined and put in writing because a similar level of trust does not exist.

Depending on the associated costs, feeding back individual research findings seems a proportional response to allowing one’s data to be used for secondary purposes. However, one might just as easily argue that a statement of gratitude, or the feedback of aggregate results, or simply the benefits of progress in medical research one receives as a member of society, would also be a proportional benefit. Indeed, one might argue that participation in research ought to be altruistic, or reflect a sense of social solidarity, and there should be no expectation of reciprocity. Even if we accept a general reciprocity obligation on the part of secondary researchers or biobanks, this does not seem to specifically require the feedback of individual research findings.

However, because there is no contact between secondary researchers and data contributors, it is difficult to see how reciprocity obligations could obtain between them at all. First, the data contributor does not give their data to any particular researcher, so it is not clear why reciprocity would require that secondary researchers provide a benefit to the specific data contributors whose data was used (rather than to the wider community). Second, there is no reason for the data contributor to trust that their contribution will be reciprocated by an individual secondary researcher—they are completely unknown to each other—and thus, there could be no corresponding obligation on the part of the researcher.

One might respond that it is in virtue of being a representative of a trusted social institution that the secondary researcher is trusted, rather than in virtue of any personal relationship between the researcher and data contributor. The obligation of reciprocity would then exist between the data contributor and the research enterprise, reflecting a sort of social reciprocity where benefits are circulated through the larger community. The reciprocity obligation could be satisfied by ensuring that individual research endeavours (aim to) provide a proportional benefit back into the community, such as through improved care.

The reciprocal obligations of biobanks to data contributors are slightly different. Many biobanks aim to rerecruit data contributors for multiple studies over time, and cultivate a relationship between the biobank and individual contributors. Even if a secondary researcher making use of the data does not have a duty of reciprocity to a data contributor, one could argue that the biobank itself might have such a duty. However, it is not clear that feeding back individual research findings is the best way to satisfy this duty, if it exists. Given the uncertain benefit of individual research results and incidental findings, one could argue that having such findings disclosed is *not* the best way to reciprocate the actions of data contributors. A further consideration is the potential impact of regularly feeding back individual research findings on the wider health system. A large influx of patients seeking consultations with their physicians concerning individual research findings could place a significant strain on the health system, and individual physicians may lack the expertise to interpret research findings.^11^ This would present a problem of distributive justice. Imposing such a burden on the wider community certainly counts against a reciprocity-based obligation to feed back individual findings.

### Professional obligations

Rather than appealing to general moral obligations, a different approach is to argue that that there are moral norms which apply in virtue of the relationship between researcher and data contributor. For example, Miller *et al* 35 argue that researchers engage in a ‘professional’ relationship with data contributors, which grounds an obligation to disclose incidental findings. They claim that as a professional, a researcher has privileged access to private information about the data contributor, and the expertise to identify its potential significance, which triggers an obligation to respond. They provide an example of a plumber and homeowner: if a plumber discovers evidence of a termite infestation while performing their contracted work, they have a responsibility as professionals to disclose this information to the homeowner.

It is not clear from Miller *et al's* account what it is about ‘professional’ relationships that generate certain moral obligations. The fact that professionals are given access to private information and have the competence to identify its potential significance is not sufficient to generate an obligation to disclose this information. Suppose a lawyer discovers some information that would benefit client A, while in a negotiation involving client B and another party. We would not say that the lawyer has a professional obligation to disclose the information to A, even though they are in a relationship, and the lawyer has expertise and access. Rather, the lawyer ought to declare a conflict of interest in relation to A (and possibly B as well). The mere fact that one is a professional does not entail obligations of beneficence with respect to private information. Therefore, the fact that a researcher is a professional doesn’t entail that they have obligations of beneficence with respect to private information. If they do have such obligations, it is because care of the information has been entrusted to them, and there is no conflict with other obligations.

The role of entrustment is implied in the plumber example; the homeowner is entrusting the plumber to look after a certain range of ‘home-care’ interests. (If the homeowner had explicitly told the plumber to only look at the water pipes—thus circumscribing the home-care interests entrusted to those concerned with plumbing and not wood framing—we would not think that the plumber had an obligation to tell the homeowner about termite damage). However, Miller *et al* also state that ‘unlike the physician, the investigator has not undertaken to act in the subject’s best interests when entering into the relationship; she has not taken on a fiduciary role.’^35^ If this is the case, why does the researcher, as a professional, have an obligation to feed back individual research findings? Miller *et al* have not provided any justification for this.

In any case, no professional relationship can be plausibly said to exist between data contributors and secondary researchers. Miller *et al* suggest that there must be at least some kind of acquaintance between the parties for a professional relationship to exist. They state: ‘when A and B are strangers…the fact that A detects a potential problem pertaining to B does not give rise to an obligation [to provide individual research findings].’^35^ Secondary researchers and data contributors have no contact or interaction, and so they are not in a professional relationship.

Conversely, the relation between data contributors and biobanks could be considered a ‘professional relationship’. During the recruitment phase, when data and samples are being collected, members of the biobank interact directly with the data contributor. They might also share the results of a data contributor’s physical examination (eg, blood pressure, bone density, visual acuity, lung function), as part of the face to face interaction, further establishing the existence of a relationship between the parties. Through this interaction, the biobank can also set the terms for the relationship in the future, including by informing data contributors that their data will be deidentified and shared only with approved secondary researchers, but also that any research findings relating to their individual data (eg, presence of a certain genotype) will not be fed back to them. (This is the model adopted by UK Biobank). Thus, the nature of this relationship is established as one of non-interaction. In this context, the most plausible obligations of the biobank (and secondary researchers accessing its data) is to ensure that contributor data remains secure and deidentified.^42^ The nature of this relationship—whether we call it ‘professional’ or otherwise—does not seem to necessitate the feedback of individual research findings.

### The partial entrustment model

The partial entrustment model provides a more developed account of the obligations of primary researchers to participants, by arguing explicitly that researchers have certain care responsibilities arising from the participant’s waiver of their rights.^43 44^ In primary research, researchers obtain permission to access private information through the informed consent process, and in doing so take on responsibility for protecting those aspects of a participant’s health which are revealed by exercising this access, including providing ancillary care.

In many respects, the partial entrustment model seems to apply to secondary research. In order for secondary researchers to have access to (reidentifiable) contributor data, the data contributors will at some point have granted permission for the secondary use of their data, either to the primary researchers who collected it, or to the biobank. Insofar as the data contributors have granted access to their information to secondary researchers—although indirectly—these researchers accrue some obligations of ancillary care.

One concern with the application of this model to secondary researchers pertains to the strength of a secondary researcher’s ancillary care obligations. On the partial entrustment model, the strength of a data contributor’s claim to ancillary care (and the strength of the researcher’s obligation to provide it) is determined by several factors: the vulnerability of the contributor, their dependence on the researcher, the level of engagement between them, the gratitude the researcher owes, and the cost of care.

A secondary researcher’s unfamiliarity with the data contributor is likely to diminish their ability to ascertain the contributor’s vulnerability or dependence, and also implies no engagement between them. Allowing their data to be used for secondary research may result in an increased risk to privacy for the data contributor, but it is unclear based on this model if the gratitude owed by the secondary researcher as a result is cumulative with that owed by the primary researcher. Finally, the added logistical challenges of reidentifying and contacting contributors in secondary research is likely to result in increased cost, and thereby weaken the ancillary care obligations of secondary researchers.

A deeper concern with the application of the partial entrustment model, however, is the important role of informed consent in fixing the scope of a researcher’s duties of ancillary care. According to the model, the scope of a researcher’s ancillary care obligations is determined by the nature of the study; researchers are only entrusted to protect those health interests which come to light in carrying out study procedures. In the case of primary research, these study procedures are conveyed to the participant as part of the informed consent process. By knowing what kind of data will be collected, but also how it will be used, the participant entrusts certain aspects of their health to the researcher. While the nature of the data that might be shared with secondary researchers in the future can be conveyed to the participant/data contributor at this time, how it will be used, in any specific sense, cannot. The upshot of this is that the disclosure of individual research findings may or may not be the kind of information which a contributor entrusts to a secondary researcher to manage responsibly, but there is no way for the secondary researcher to ascertain this information, in the absence of some contact with the contributor.

Finally, the partial entrustment model is ill suited for data-driven research, such as the development of machine-learning algorithms, which forms a significant proportion of current health research using secondary data. It is difficult to see how care obligations might apply to researchers using health data outside of a care context. For example, what sorts of care obligations would a data scientist using chest CT scans to train a machine-learning algorithm have to the contributors of this data, assuming it was possible for the data to be reidentified? With respect to incidental findings, a data scientist would not have the necessary expertise to identify potential abnormalities in a scan. Would they be required to obtain expert analysis of the scans? On a strong interpretation of the ancillary care model—and depending on the associated costs—one could seemingly argue in the affirmative. Even setting aside the logistical challenges, we argue that such an obligation is too demanding. While the partial entrustment model may provide justification for a primary researcher’s responsibility to disclose certain kinds of individual findings, this justification does not apply to secondary researchers.

## The obligations of biobanks and secondary researchers

We have argued that none of the standard justifications for an obligation to disclose individual research findings in primary research apply adequately to the case of secondary research. In some cases, this is because there are features of secondary research which distinguishes them from primary research, and renders the standard justifications for feedback inapplicable. In other cases, there are weaknesses in the arguments themselves, and so they fail to justify the feedback of individual results in secondary research. Nevertheless, these accounts do illuminate important considerations. Specifically, the nature of the relationship between secondary researchers and data contributors is an important factor in determining an appropriate strategy for managing individual findings.

When data contributors agree to allow their data to be used for secondary research, they are indirectly granting future researchers discretionary power over their data; how the data are analysed and processed, what ends it will be used for, and how it will be protected and stored. Data contributors have certain expectations for the appropriate use of their data, regardless of the particular secondary researcher who accesses it. In exchange for contributing to the public good of research, they reasonably expect that their interests will be protected, through the establishment and enforcement of adequate regulations for the conduct of research in general (eg, the Declaration of Helsinki, The US ‘Common Rule’) or the use of secondary research data (eg, the GDPR).

These regulations and guidelines help to define the standards for scientific and ethical acceptability of research using secondary data and provide the context in which any relationship between individual researchers and data contributors exists. Secondary researchers have a moral—and legal—obligation to adhere to these standards. By doing so, secondary researchers ensure that the agent-neutral interests of data contributors, such as the interest in privacy and security of their data, are protected. It is a further question whether secondary researchers have additional obligations to data contributors, in virtue of a relationship between them as individuals. We argue that they do not.

We stated above that data contributors indirectly grant secondary researchers discretionary powers over their data. In granting these powers—even indirectly—data contributors necessarily face the risk that this power will be misused. Indeed, it is because of this potential vulnerability that strong oversight and regulation by the state is justified. Within these regulatory boundaries, however, secondary researchers can also affect the interests of data contributors. For example, a secondary researcher might conduct research which risks marginalising a certain social group. The appropriate use of their discretionary powers will depend on the nature of the interests entrusted to them by data contributors.

Secondary researchers are granted access to contributor data for the purposes of conducting research for the benefit of society. The interests entrusted to them are to ensure that data is used for this purpose, and that contributor privacy is protected. It is not to protect or promote their health. Thus, we argue that the appropriate use of their discretionary power is limited to these purposes, which does not require disclosing individual findings.

A similar argument applies to biobanks themselves. The obligation of the state to protect the interests of data contributors, as explicated in ethical regulations, requires biobanks to adhere to strict requirements regarding the acquisition, storage and distribution of contributor health data. However, unlike secondary researchers, biobanks might be argued to have a relationship with data contributors that generates some sort of duty of care. Data contributors directly entrust biobanks with discretionary powers pertaining to the collection, storage, and distribution of their data, as well as further discretionary powers necessary for acquiring this data (eg, the power to conduct tests, collect samples, and curate data). Biobanks may also have access to a range of identifiable contributor health and demographic data. Moreover, because biobanks may follow up with contributors periodically for longitudinal studies, or recontact them to participate in further testing, it is plausible that data contributors may come to view their relationship with the biobank as one in which they have entrusted at least some of their health interests (even if the biobank is explicit that they are not providing care to contributors).

Nevertheless, we argue that even if the relationship between biobank and data contributor is a trust relationship, it does not follow that there is an obligation for biobanks to disclose individual research findings to data contributors. Trust relationships give rise to a range of obligations reflecting the structure of the relationship (ie, where one party entrusts the other with power over a significant practical interest), which may include a duty of care.^45^ How these duties are specified depends on the interests entrusted. The purpose of the relationship between data contributors and biobanks is the conduct of health research that is broadly in the public interest. When a data contributor provides their data to the biobank, they are entrusting them with the power to use this data to conduct socially beneficial research. The biobank’s ‘duty of care’ amounts to an obligation to act in a manner that meets this trust. For example, they must not allow contributor data to be used for research that might be considered socially harmful.

The disclosure of individual research findings, on the other hand, constitutes a legitimate exercise of the discretionary power of the biobank only insofar as protection of the contributor’s health interests has been entrusted by the data contributor. In such a case, feedback would occur in accordance with what the biobank judges to be consistent with the relevant health interests of the data contributor. Thus, if a biobank imposes a blanket policy of non-disclosure of individual research findings, this is consistent with their obligations to data contributors if the biobank has made clear that data contributors are only entrusting the protection of their data to the biobank, and not entrusting any health interests. Conversely, a biobank may offer to accept responsibility for certain health interests of data contributors. If contributors choose to entrust these interests, the biobank’s duty of care changes accordingly—and may include an obligation to feedback individual research findings—because the nature of the relationship has changed.

Thus, while there is no moral obligation for biobanks to feed back individual research findings, there is also no moral obligation to withhold them. The obligations of the biobank depend on the nature of the relationship that has been established with data contributors. This leads to the possibility of offering to feed back individual research results for pragmatic reasons, such as a desire on the part of the biobank to increase enrollment, or because participants express a desire for them. For example, the Icelandic biopharmaceutical company deCODE, which has collected genomic and other biomedical data from nearly two-thirds of the adult population of Iceland, provides feedback of select individual research results through a secure web-based portal.^46^ Participants can choose to access their genetic status regarding a pathogenic variant of the *BRCA2* gene, and genetic counselling is made available in positive cases. Insofar as this opportunity for feedback is understood as a ‘bonus’ of participation, it does not change the nature of the relationship between the biobank and data contributors. On the other hand, providing some individual research results might be interpreted by data contributors as the biobank accepting responsibility for their health interests.

In either case, it is critical that the nature of the relationship and the interests being entrusted be clearly conveyed to prospective data contributors. Unlike the role of the physician, which has been developed and refined over centuries and whose duty of care is well understood by most patients, the role of the biobank is less familiar. It is possible that some data contributors may believe themselves to be entrusting their health interests to the biobank, and thereby expect the feedback of individual research findings. Clear communication about the role of the biobank is key to ensuring that the expectations of data contributors are aligned with the obligations of the biobank.

## Conclusion

The management of individual findings in research presents numerous ethical challenges for researchers, particularly in the context of secondary research. We have argued that the standard justifications for an obligation to disclose such findings in the context of primary research do not straightforwardly apply to secondary research, and an alternative conception is needed. We have attempted to provide a sketch of what these obligations might look like, for both secondary researchers and biobanks. We argue that the extent to which a relationship exists between data contributors and researchers, and thus the extent to which a data contributor entrusts certain discretionary powers to the researcher, influences the obligations of the researcher (or biobank), including the provision of individual research findings.
